# Supplementary material for: Comparative multiomics analysis of cell physiological state after culture in a basket bioreactor
Source: Sci Rep. 2022 Nov 23;12:20161. doi: 10.1038/s41598-022-24687-4 (PMC9686226; doi:10.1038/s41598-022-24687-4)
Supplement: Supplementary file 1 — Supplementary Information 1. [file 41598_2022_24687_MOESM1_ESM.zip › raw data/Transcriptomic raw data/1.QC/2.GC/TC1_2_GC.pdf]

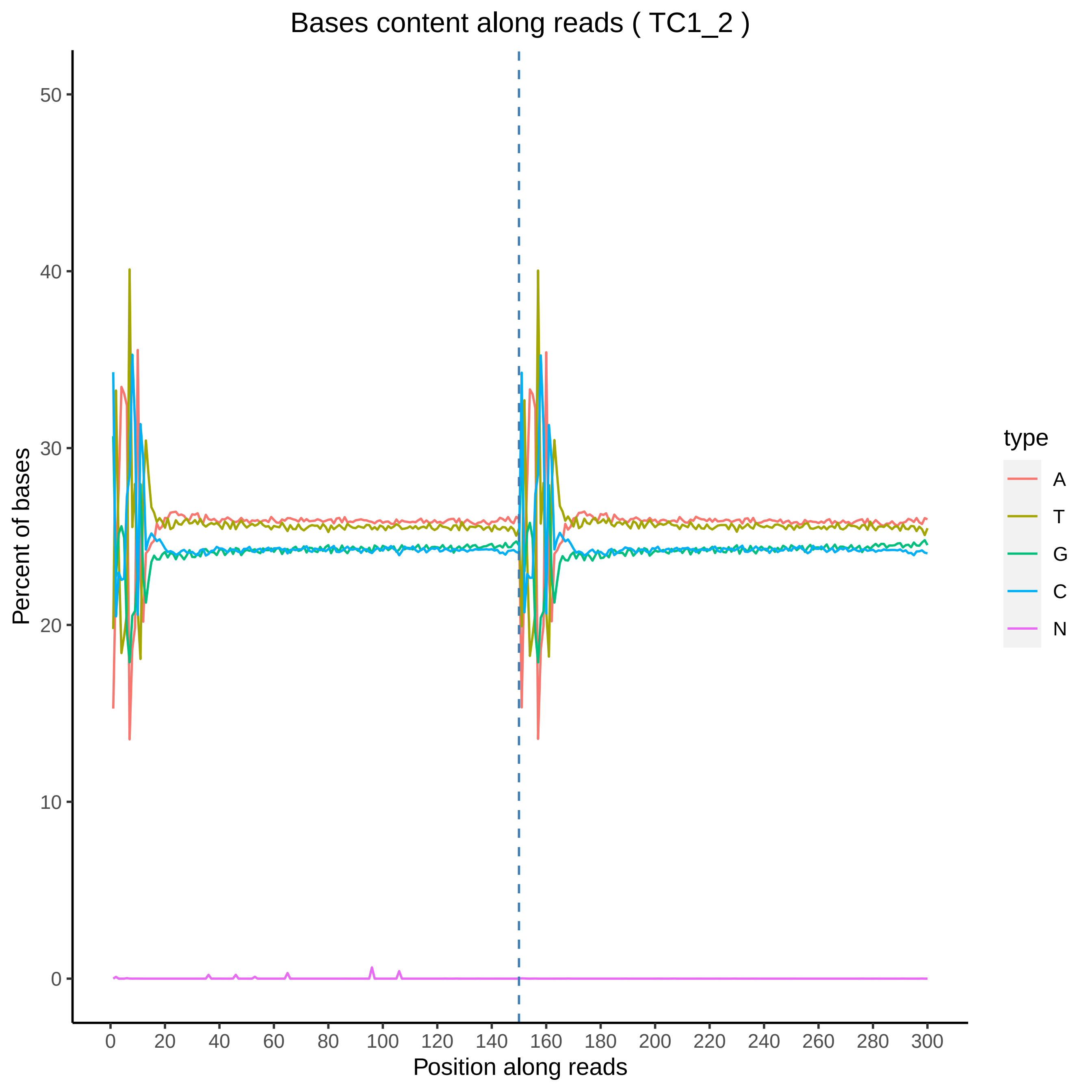
Bases content along reads (TC1_2)
Percent of bases
0
10
20
30
40
50
Position along reads
0
20
40
60
80
100
120
140
160
180
200
220
240
260
280
300
type
A
T
G
C
N
